# Initial psychometric evaluation of the European Portuguese version of the Lincoln Canine Adaptability Resilience Scale

**DOI:** 10.1371/journal.pone.0339725

**Published:** 2025-12-31

**Authors:** J. C. Alves, Ana Santos, P. Jorge, T. Mendonça

**Affiliations:** 1 Divisão de Medicina Veterinária, Guarda Nacional Republicana (GNR), Lisbon, Portugal; 2 Faculty of Veterinary Medicine, Lusófona University, Lisbon, Portugal; 3 Centro de Ciência Animal e Veterinária, Lusófona University, Lisbon, Portugal; 4 I-MVET, Faculty of Veterinary Medicine, Lusófona University, Lisbon University Centre, Lisbon, Portugal; 5 MED – Mediterranean Institute for Agriculture, Environment and Development, Instituto de Investigação e Formação Avançada, Universidade de Évora, Évora, Portugal; Federal University of Santa Maria: Universidade Federal de Santa Maria, BRAZIL

## Abstract

**Objective:**

Dogs face a variety of stressors in their lives, either as companion or as working animals, from novel situations and challenges, to visits to the vet, kenneling, or transportation. Some individuals have personal characteristics that allow them to cope with adversity and develop positive adaptations. The Lincoln Canine Adaptability Resilience Scale (L-CARS) was developed to assess this ability. This study aimed to validate a European Portuguese version of the Lincoln Canine Adaptability Resilience Scale.

**Patients and methods:**

Information from 182 dogs was collected. The English version of the L-CARS was translated into Portuguese, and this version was back-translated until a unified version was obtained. Canine handlers, native Portuguese speakers, completed a copy of the translated Portuguese version of the L-CARS. Validity testing was performed with the Kaiser-Meyer-Olkin measure of sampling adequacy. Extrated values were assessed based on Eigenvalue and scree-plot analysis. Internal consistency was tested with Cronbach’s α. The correlation between items was determined using Spearman’s rank correlation coefficient.

**Results:**

Kaiser-Meyer-Olkin factor analysis was 0.83. The factor analysis model identified five factors with an eigenvalue greater than 1, accounting for 67.56% of the variance. Commonalities ranged between 0.86 and 0.29. Cronbach’s α was 0.81. All components showed positive correlation with the overall score (p < 0.01 for both).

**Conclusions:**

The study presented criterion and construct validity of the European Portuguese version of the L-CARS. It is a step in providing a broader Portuguese-speaking population and clinicians with a validated and accessible tool to evaluate patients. Further studies are required.

## Introduction

From a psychological perspective, resilience is generally regarded as the ability to “bounce back” following adversity or trauma [1,2]. Dogs face a variety of stressors in their lives, either as companion or as working animals, from novel situations and challenges, to visits to the vet, kenneling, or transportation [3–5]. The ability to cope with these stressors is crucial for all dogs, particularly working dogs. The inability to “bounce back” following adversity or trauma can lead to behavioural problems, a common reason for early retirement in working dogs [6]. Some individuals have personal characteristics that allow them to cope with adversity and develop positive adaptations [7]. Several approaches have been tried in puppies to improve their resilience in the face of future challenges [8,9].

The Lincoln Canine Adaptability Resilience Scale (L-CARS) is a psychometric tool for measuring resilience in dogs. It comprises 14 items, graded on a 5-point Likert scale (from 5, “Strongly agree” to 1, “Strongly disagree”). The L-CARS shall consist of 2 principal components (PC): “adaptability/behavioural flexibility” (PC1), and “perseverance” (PC2) [3]. It has been shown to have predictive validity for behavioural problems, which are expected correlates [3].

Translating an existing instrument has several advantages, the main one being the ability to leverage all the scientific work done to support the instrument, even in other translated versions [10]. Nevertheless, when a translation is performed, the new version must undergo psychometric testing [11]. This allows for the assessment of whether several properties are kept in the new version [12], and are not influenced by cultural or vocabulary differences [13–15]. A primary evaluation is conducted to determine whether the scale evaluates what it aims to evaluate [16,17]. For this purpose, a group of experts performs the assessment, thereby ensuring face validity. Behavioural traits cannot be directly observed, as pain [18]. For that reason, construct validity is evaluated [18,19], through factor analysis. Cronbach’s α is a common measure of internal consistency [16,20]. Additionally, it can also be determined if a relationship exists between two measures, which constitutes convergent validity [18].

This study aimed to validate a European Portuguese version of the Lincoln Canine Adaptability Resilience Scale, in a first step to allow its availability to Portuguese speaking populations, a language spoken by 261 million people around the world [21], as a primary language. The hypothesis was that the Portuguese version would show the reliability and validity documented in the English version.

## Materials and methods

The study protocol was approved by the ethical review committee of the Faculty of Veterinary Medicine, Lusófona University (Comissão de Ética e Bem Estar Animal – CEBEA, approval nº 30/2025) and complied with the relevant institutional and national guidelines for the care and use of animals. The ARRIVE guidelines for reporting were followed. Written informed consent was obtained from the Institution responsible for the animals. Written permission to translate the L-CARS into Portuguese was obtained from the copyright holder.

The procedure for the translation process followed existing guidelines [22]. Two bilingual researchers, native-speaker of the target language, performed the initial translation of the English version of L-CARS into Portuguese. These versions were compared by a third independent researcher, who compared the two versions. After a unified translation was reached, it was translated back into English by another bilingual translator, with native-level English proficiency. The two English versions were compared for accuracy until a final version was reached [23].

The L-CARS comprises 14 items, graded on a 5-point Likert scale: 5 (“Strongly agree”), 4 (“Mainly agree”), 3 (“Partly agree, partly disagree”), 2 (“Mainly agree”), and 1 (“Strongly disagree”). For items 1, 3, 6, and 7, the score should be reversed, i.e., score of 5 becomes 1; 4 becomes 2; 3 remains 3; 2 becomes 4; 1 becomes 5. There is also a possibility for a “not applicable/don’t know” response. The L-CARS comprises 2 principal components (PCs): PC1 “adaptability/behavioural flexibility” (11 items), and PC2 “perseverance” (3 items) [3]. The score for each PC is obtained with the following formula: Score = sum of item scores/ (number of items × 5)Score = sum of ansewered items score/ (number of items × 5). The “normal” range of scores is 0.72–0.92 for PC1, and 0.64–0.98 for PC2 [3]. The original version of the L-CARS is available online (https://ipstore.lincoln.ac.uk/product/lincoln-canine-adaptability-scale-l-cars). The Portuguese version of the L-CARS is shown in Table 1.

**Table 1:** Portuguese version of the Lincoln Canine Adaptability Resilience Scale (L-CARS).

		Concordo fortemente	Concordo maioritariamente	Concordo parcialmente, discordo parcialmente	Discordo maioritariamente	Discordo fortemente	N/A ou não sei
**1**	Se algo assustou o meu cão, ele ficará nervoso por regressar àquele local durante muito tempo após o evento.						
**2**	Se o meu cão tiver uma má experiência com outro indivíduo (cão ou pessoa), ele esquece depressa, e não fica agarrado a isso.						
**3**	Se algo sobressaltasse ou assustasse o meu cão, ele ficaria agitado durante muito tempo após o evento.						
**4**	O meu cão não fica chateado facilmente.						
**5**	O meu cão geralmente enfrenta situações stressantes com calma.						
**6**	O meu cão por vezes parece fora de controlo sem razão aparente.						
**7**	Se outro cão tem uma reação negativa a uma coisa, é provável que o meu cão também fique afetado negativamente.						
**8**	O meu cão normalmente gosta de qualquer coisa que seja nova ou incomum, isto é, objectos, animais ou qualquer coisa que nunca tenha visto antes.						
**9**	Consideraria que o meu cão é facilmente adaptável, isto é, capaz de se adaptara qualquer situação.						
**10**	Se qualquer coisa inesperada acontecesse e as circunstâncias da minha vida mudassem, sei que o meu cão conseguiria lidar com isso.						
**11**	Acredito que o meu cão é resiliente, isto é, capaz de lidar com, recuperar de e/ou adaptar a adversidades ou mudanças.						
**12**	O meu cão dá sempre o seu máximo, mesmo quando a tarefa é difícil.						
**13**	O meu cão vai persistir mesmo quando não consegue fazer algo imediatamente, isto é, quando aprender um novo truque ou tenta resolver um problema, etc.						
**14**	O meu cão aprecia desafios, isto é, aprender novas habilidades, encontrar objetos escondidos, resolver uma tarefa difícil.						

Para cada uma das seguintes afirmações, por favor selecione aquela que descreve como acha que o seu cão se comporta na situação, presentemente. Sabemos que pode ser difícil, mas tente pensar como o cão se comportaria no geral, naquele cenário.

Se o seu cão nunca esteve perante a situação descrita, ou se acha que não consegue prever com precisão o comportamento do cão neste contexto, por favor selecione a opção “N/A ou não sei”.

A digital version was sent via email to the canine handlers of the Portuguese Gendarmerie. To be included in the study, the handlers had to have an active working dog in their care. All canine handlers must be at least 18 years old. The email included a summary of the project and an estimated time for completion. Handlers were requested to read the information and select whether they agreed to proceed to the scale, thereby constituting informed consent.

The animal population was composed of police working dogs of the Portuguese Gendarmerie. They were individualy housed in similar kennels, in different locations around Portugal. In assition to a training and working schedule conducted alongside each animal’s specific handler, according to the type of work conducted, the animals have alse the controlled contacted with other working dogs, belonging to the same unit.

The Kaiser-Meyer-Olkin measure of sampling adequacy was used to perform factor analysis and to test validity. Values over 0.6 were considered adequate [20]. Eigenvalue and scree-plot analysis were used to evaluate extracted values. A varimax-rotated model of factor analysis was used for the item loading on the extracted components, with a value of 0.4 limited to the considered communality [17]. The correlation between items was determined using Spearman’s rank correlation coefficient. A significance level of p < 0.05 was set. The internal consistency of observed results was evaluated with Cronbach’s α. A reliable value was set at 0.8 or more [19]. The commercial software IBM SPSS Statistics, version 27, was used to analyze the data.

## Results

One hundred and eighty-two responses to the questionnaire were obtained. The Police working dogs included in the sample had a mean age of 4.3 ± 3.2 years, with 117 males and 65 females, with the following breeds distribution: Belgian Malinois Shepherd Dogs (n = 95), German Shepherd Dogs (n = 35), Dutch Shepherd Dog (n = 21), Labrador Retriever (n = 12), and Other (n = 19). Regarding specific mission, 70 were patrol dogs, 58 were employed in drug detection, 21 in search and rescue, 20 in explosive detection, 5 in tactical interventions, 4 in poison detection, 3 in tobacco detection, and 1 in dog-assisted therapies.

**Fig 1:**
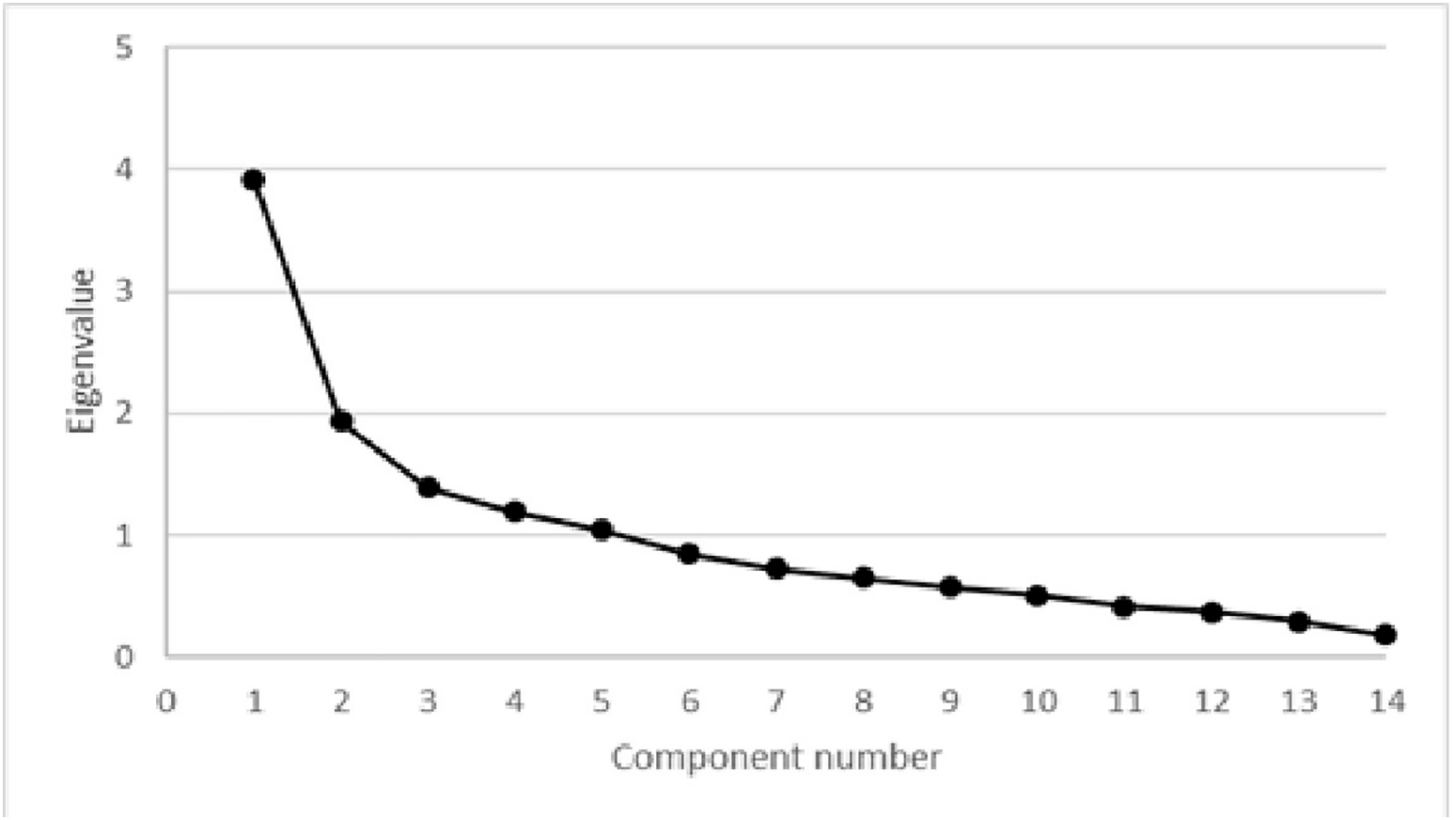
Scree plot of factor analysis of the portuguese version of the Lincoln Canine Adaptability Resilience Scale (L-CARS). Five factors had Eigenvalues >1, and a discernible “shoulder” can be observed in the graphic. The five factors accounted for 67.56% of the variance.

Kaiser-Meyer-Olkin factor analysis was 0.83. As all values were above 0.8, factor analysis was conducted. The varimax-rotated factor analysis model identified five factors with an eigenvalue greater than 1, accounting for 67.56% of the variance. The remaining factors have eigenvalues ranging from 0.84 to 0.18. The scree-plot confirmed the retention of the 5 factors with an eigenvalue greater than 1 (Fig. 1). Based on the varimax-rotated solution, the loading for the items was performed. Commonalities ranged between 0.86 and 0.29. Cronbach’s α was 0.81. A moderate to high correlation was observed between the PCs and the overall score (p < 0.01 for both), but not between the PCs themselves. The correlation between PCs is presented in Table 2.

**Table 2:** Correlation between principal components (PC) of the portuguese version of the Lincoln Canine Adaptability Resilience Scale (L-CARS).

		Overall	PC1	PC2
**Overall**	rs	1	0,742	0,609
	Sig.		p < 0.01	p < 0.01
**PC1**	rs	0,742	1	0,05
	Sig.	p < 0.01		0,947
**PC2**	rs	0,609	0,05	1
	Sig.	p < 0.01	0,947	

## Discussion

The results demonstrate that the Portuguese version of the L-CARS exhibits adequate internal consistency and construct validity, comparable to those reported for the original English version³. This new translation, now available in European Portuguese, is a step toward increasing the availability of this validated instrument to a broader population, enabling its use in clinical and research settings [18].

In the process of psychometric testing, factor analysis is commonly used to assess construct validity, and internal consistency is assessed through Cronbach’s α [16,24,25]. Evaluating validity supports the theoretical construct that the instrument is measuring what it is supposed to measure [16,24]. The results of the factor analysis and scree-plot analysis extracted 5 components with eigenvalues greater than 1 for the Portuguese version of the L-CARS. These results differ from the English version, which identified 2 components [3]. It is not uncommon for different populations to have different factor analysis results. The characteristics of the population considered for evaluating the instrument influence the results obtained. The English version was validated in a population from several countries, including the United Kingdom, the United States of America, Ireland, Canada, New Zealand, and others. It also featured multiple breeds and cross-breed dogs. For the European Portuguese version, only a few, relatively homogeneous breeds were included. All were working dogs, housed in similar conditions and with a shared training background. Additionally, the questionnaire was completed by a group of active canine handlers, who should be more aware of different behaviours and traits [26,27]. This approach is of interest at a first stage, as individual variability is reduced in an animal population with these characteristics. Having trained canine handlers as the human proxy lickely also helps in the accurate completion of the questionnaire. However, it raises the need for testing in a broader population.

The results obtained through factor loading were also supported by the inter-item correlations and Cronbach’s α [18]. The results observed with Cronbach’s α may have been influenced by the homogeneous sample with a low overall variance of item scores [28], since the results obtained with Cronbach’s α may vary for different populations. A correlation was observed between the two components and the overall score. This finding supports the idea that the components are related, but are surely not redundant, as there no correlation between the two.

This first study was conducted in a controlled European Portuguese-speaking population. Future perspectives for further validation of the Portuguese version of the L-CARS include validating the scale in a broader population and enrolling more animals with diverse characteristics, including various breeds and origins (e.g., rescue shelters, companion dogs, behavior clinics, and others). Opening the use of the L-CARS in different settings can also lead to the opportunity to evaluate how it performs to assess response to interventions, and, in line with what was conducted with the original version.

## Conclusions

In this study, we performed the initial validation of the Portuguese version of the L-CARS and its validity in the Portuguese language. Further studies are required to determine if the present results can be replicated across samples and clinical settings.
